# Complementing Onsager’s Conductivity Theory by Grotthuss Mechanism Mitigation via Ion-Induced Depletion of Hydrogen-Bond-Donating Water

**DOI:** 10.1021/acs.jctc.6c00901

**Published:** 2026-07-04

**Authors:** Benjamin Janotta, Maximilian Schalenbach, Hermann Tempel, Wei-Hsuan Hung, Rüdiger-A. Eichel

**Affiliations:** † Fundamental Electrochemistry (IET-1), Institute of Energy Technologies, 28334 Forschungszentrum Jülich, Wilhelm-Johnen-Straße, 52425 Jülich, Germany; ‡ Institute of Materials Science and Engineering, 34911 National Central University, No. 300, Zhong-da Rd., Zhongli District., Taoyuan City 320, Taiwan, ROC; § Institute of Physical Chemistry, RWTH Aachen University, 52062 Aachen, Germany; ∥ Faculty of Mechanical Engineering, RWTH Aachen University, 52056 Aachen, Germany

## Abstract

Modern Onsager-type conductivity models enable low-uncertainty predictions for electrolytes up to a few moles per liter while using a small set of physical parameters. However, their application to acids and bases results in significant deviations from measurement data and has thus remained limited. Here, we present a model that explicitly describes the molar conductivity due to the Grotthuss mechanism and its dependence on the ion concentration, which is not considered in Onsager-type models. In line with the literature, our model considers a single rate-determining step for a successful Grotthuss mechanism that depends on the ability of water molecules at a reaction site to accept or clear hydrogen bonds. Water in the hydration shell of ions loses this ability due to strong ion-dipole forces, leading to a decrease in Grotthuss conductivity as the ion concentration increases. The two introduced model parameters enable excellent agreement between the model and measurement data for strong acids and bases, while their values are physically explainable. The model reproduces the experimentally observed maxima in conductivity data of acids and bases and explains their physical origin as a result of the declining Grotthuss conductivity.

## Graphical abstract

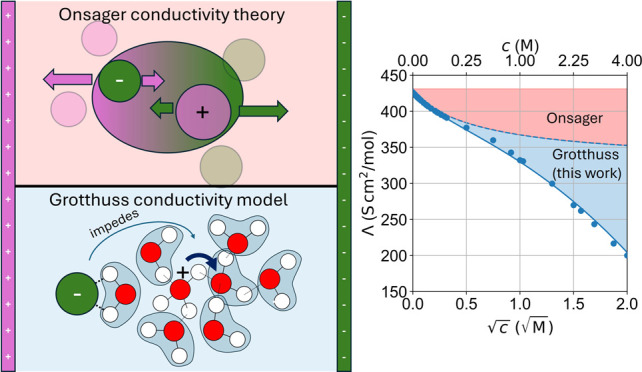

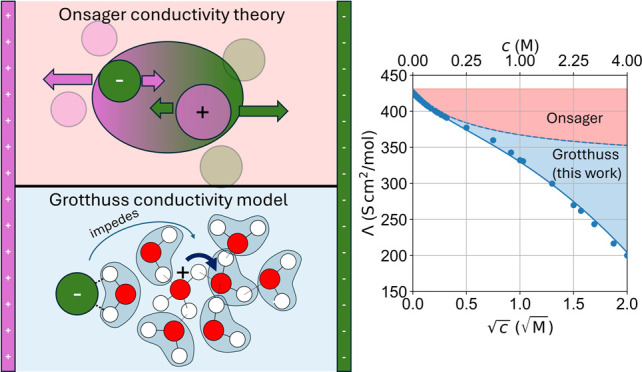

## 1 Introduction

Despite more than two centuries of research, the origin of electrolytic conductivity remains a subject of active investigation and debate.^1–5^ In 1806, Grotthuss^6^ first described the idea behind a mechanism now known to contributeto conductivity in pure water: the Grotthuss mechanism. Roughly 80 years later, Arrhenius^7–9^ laid the foundations of electrolytic dissociation, explaining the conductivity of dissolved salts phenomenologically. The first physically sound model explaining the decline of the molar conductivity with ion concentration quantitatively was developed by Onsager and Fuoss around 1930.^10,11^ Their work extends the Debye–Hückel theory,^12,13^ so the first model successfully approximating distribution functions in dilute electrolytes, with models for electrophoretic (hydrodynamic retardation) and relaxation (electrostatic interaction with the asymmetric ionic atmosphere) effects derived from considerations of continuum mechanics. The so-called Debye–Hückel-Onsager theory (short: Onsager theory) quantitatively explains the conductivity of electrolytes up to concentrations of about 0.1 M, a limit that also constrains the Debye–Hückel theory.

In the literature, two categorically different development strategies were followed to improve the Onsager theory: (1) Refining the electrophoretic and relaxation effects,^14^ and (2) redeveloping the model based on a refined electrolyte structure.^15^ Models categorized by the second approach were, for example, developed for the Mean Spherical Approximation,^16^ which overcame significant drawbacks of the Debye–Hückel theory.^17,18^ Consequently, the conductivity of numerous binary and multi-ion electrolytes could be modeled up to few moles per liter, gaining practical relevance for these models.^5,15,19^ However, in the literature, few models are presented aiming to describe acids and bases accurately at practically relevant concentrations.^20,21^

Acids and bases exhibit extraordinarily high conductivity, which is ascribed to the Grotthuss mechanism.^6,22^ The Grotthuss mechanism enables fast transfer of the charge ascribed to a proton (or a hydroxide ion, respectively) by tunnelling through water molecules, while the respective atoms move little. In the last decades, the Grotthuss mechanism in water and other electrolytes was extensively investigated using computational and experimental methods.^4,23–26^ However, modern molecular dynamics simulations still struggle to predict the conductivity of simple binary electrolytes quantitatively,^27^ while (to the best of our knowledge) models accurately quantifying the macroscopic effects of ion concentration on the Grotthuss mechanism have not been proposed.^28^ This deficiency of theoretical considerations explains the absence of Onsager-type models capturing acids and bases.

In this work, we present a model explaining the decreasing Grotthuss conductivity with ion concentration and apply it to strong acids and bases as an extension for an Onsager-type model. The exceptionally fast decrease of the molar conductivity of acids and bases is ascribed to an increasing number of water molecules bound in hydration shells, which decreases the probability for a successful Grotthuss mechanism. The model shows quantitative agreement with measurement data for the conductivity. Importantly, the maximum conductivities of acids and bases are accurately displayed by our model, which is not achieved with traditional Onsager-type models. Therefore, the proposed model explains a deficiency of the Onsager-type model and may consequently facilitate the development of concentration-dependent simulations with practical relevance.^29–31^

## 2 Theory and Methodology

In the following, we derive a model that captures the declining contribution of the Grotthuss mechanism to the total conductivity with increasing ion concentration (henceforth called GM for “Grotthuss model”). We consider strong acids and bases (strong denoting completely dissociated) of binary electrolytes (binary denoting only one type of cation and anion). The counterion of the respective acid or base will be considered a general ion and be denoted by X, while H^+^ and OH^–^ are denoted by A. The concentration dependences of all ions are calculated using the Onsager-type models for relaxation and the electrophoretic effect derived by Roger et al.^32^ (henceforth called OM for “Onsager-type model”) based on the Mean Spherical Approximation (see Supporting Information).

The molar ionic conductivity λ_A_ arises as a superposition of the Vehicle mechanism (λ_A,V_), so charge transfer due to mass-transfer of the respective ion, and the Grotthuss mechanism (λ_A,G_),^22,33–37^ eq 1

1 $$\lambda_{A}=\lambda_{A,V}+\lambda_{A,G}$$

The superposition implies that both processes appear independently of each other and may happen simultaneously. Figure 1 illustrates the concept and the magnitudes of the superposition in comparison to alkali metal and halide ions. The ionic molar conductivity increases from ions with small Pauling radii toward ions with larger Pauling radii (Li^+^ to K^+^ as well as F^–^ to Br^–^) due to larger hydration shells that are transported with the smaller ions (due to higher charge density).^38^ The hydration shell of H^+^ is considered to fluctuate between debated configurations like the Zundel ion^39,40^ (H_5_O_2_^+^) and Eigen ion^41^ (H_9_O_4_^+^),^22,35,42–44^ and therefore also carries a large hydration shell. Hence, λ_H^+^,V_ is assumed to follow the trend of the alkali metal ions and is thus smaller than λ_Li^+^,V_. According to Kreuer et al.,^37,45^ the upper limit of the part assigned to the Vehicle mechanism is 22% of the total molar conductivity of H^+^ (marked by the top of the black bar in Figure 1), in accordance with our assumption. This upper limit of the Vehicle mechanism is denoted by λ_A,V_^max^. Respective arguments are made for OH^–^. Accordingly, we set λ_H^+^,G_ = 314.8 and λ_OH^–^,G_ = 168.6 as reference parametrization for the Grotthuss conductivity, accounting for 90% and 85% of the limiting molar conductivity of H^+^ and OH^–^, respectively. The difference in absolute Grotthuss conductivity is explained by the tendency of the OH^–^ to reside in a stable hypercoordinated state in which its oxygen accepts four H-bonds (one more than the three-coordinated tetrahedral geometry required for proton transfer). This state is inert to proton transfer, and hopping can only occur after a thermally activated presolvation step reduces the coordination number to three.^36,46,47^

**Figure 1:**
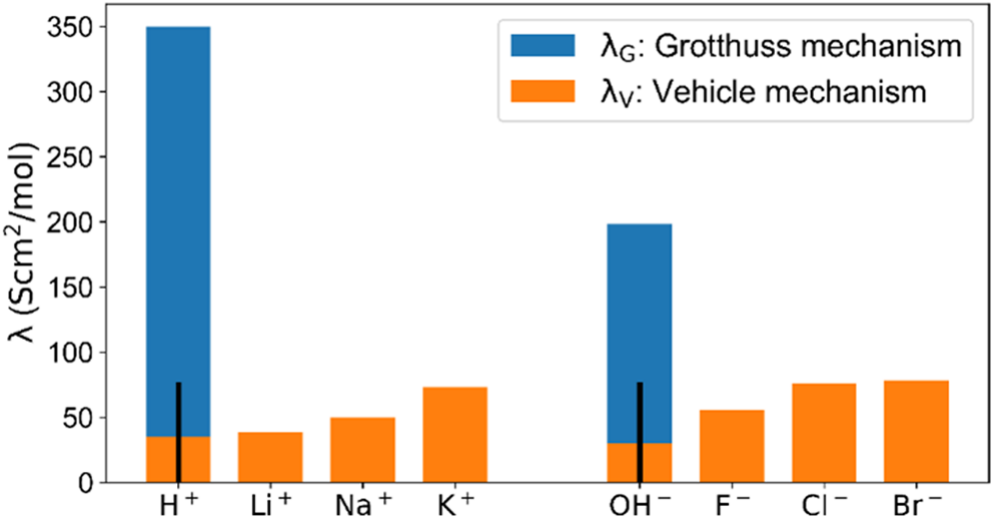
Molar conductivity of H^+^ and OH^–^ are assumed to result from the sum of the Vehicle (mass-transfer-bound) mechanism and the Grotthuss mechanism. For H^+^ and OH^–^, 90% and 85% of the conductivity at infinite dilution are assigned to the Grotthuss mechanism, respectively. These values are approximated from the trends of the alkali metal ions and the halide ions. Hence, the exact value chosen of both contributions is to some extent arbitrary. The black bars indicate intervals used for a parameter variation in Figure 4. Total ionic molar conductivity at infinite dilution from the literature.^38^

To simplify the derivation of how λ_A,G_ varies with ion concentration, we focus on the drift velocity rather than the conductivity itself. The ionic molar conductivity is proportional to the drift velocity ***v***, which itself is defined as the average velocity of ions reacting to a potential gradient **∇**ϕ (in this case, a macroscopic electric field).^45^ As ***v*** and **∇**ϕ are vector quantities, their direction is relevant. Hence, we define the normal vector ***n*** that describes the direction of **∇**ϕ, eq 2

2 $$n=\frac{\nabla \phi }{\left|\nabla \phi \right|}$$

Considering a scalar-valued conductivity, only the normal component of the charge transport in the direction of the externally applied electric field contributes to the measured conductivity of an electrolyte.^48–50^ Now, we can describe the ratio of the ionic molar conductivity at a finite concentration to the one at infinite dilution by the ratio of the (normal-directed) drift velocities, eq 3

3 $$\frac{\lambda_{A,G}}{\lambda_{A,G}^{0}}=\frac{v_{A,G}\cdot n}{v_{A,G}^{0}\cdot n}$$

The dot indicates a scalar product, the superscript 0 indicates the state at infinite dilution. Within this work, we formulate the drift velocity due to the Grotthuss mechanism as the product of an average step distance in the normal direction $\overset{®}{\Delta x}=\overset{®}{\Delta x}\cdot n$ (due to the applied electric field) and the average frequency *f̅* with which successful Grotthuss mechanisms occur. Macroscopically, this frequency may be described as a reaction rate *r*_G_ of the Grotthuss mechanism with respect to the concentration *c*_A_: *r*_G_ = *c*_A_ *f̅*. Hence, emphasizing the direction dependence, we formulate eq 4

4 $$v_{A,G}\cdot n=\frac{r_{G}}{c_{A}}\overset{®}{\Delta x}$$

In electrostatic equilibrium (zero current density), the drift velocity is zero because the average step distance is $\overset{®}{\Delta x}=0$ . During conduction, the external field is responsible for a preferential component of $\overset{®}{\Delta x}$ in the direction of ***n***. Introducing our definition of the Grotthuss drift velocity, eq 4, into eq 3 gives eq 5

5 $$\frac{\lambda_{A,G}}{\lambda_{A,G}^{0}}=\frac{\frac{r_{G}}{c_{A}}\overset{®}{\Delta x}}{\frac{r_{G}^{0}}{c_{A}^{0}}\overset{®}{\Delta x^{0}}}$$

For the reaction rate *r*_G_, we follow the state of the literature^4,22,24,51^ and assume that the Grotthuss mechanism is governed by a single rate-determining step involving a “free” water molecule (denoted by superscript “*f*”).^24,51^ We define “free” water molecules as those being able to accept (or clear) a hydrogen bond to enable a successful proton hopping. Agmon^7^ concluded that the most likely mechanism is “a hydrogen-bond cleavage, taking place in front of the moving proton, and hydrogen bond formation in its back”, where the hydrogen-bond cleavage is the rate-determining step. Figure 2a illustrates the model of Agmon^22^ (compare his Figure 2). Recently, Gomez et al.^51^ concluded that the rate-limiting step is the second of two necessary hydrogen-bond exchanges (Steps 1 and 3 in Figure 2a), preventing a fast backward hopping. Similarly, Fischer et al.^24^ concluded that “the presence of a fourth water molecule, donating a hydrogen bond to the hydronium ion at the time the excess proton leaves, results in a decreased probability for the proton to return to that host water molecule” from ab initio molecular dynamics simulations. In water, the Grotthuss mechanism is facilitated by strong hydrogen bonding of water molecules.^45^ Complementing the definition of “free”, we define “bound” water molecules (superscript “*b*”) as those that do not donate a hydrogen bond or do it at least with a lower probability than “free” water molecules. The water molecules in the first hydration shell of counterions are considered unfavorable for participation in the Grotthuss mechanism. Similarly, the water molecules in the first hydration shell of OH^–^ or H^+^ are assumed not to participate in the Grotthuss mechanisms of their own species.^22^ Figure 2b and 2c illustrate the concept that water molecules that are bound to ions cannot participate in the Grotthuss mechanism.

**Figure 2:**
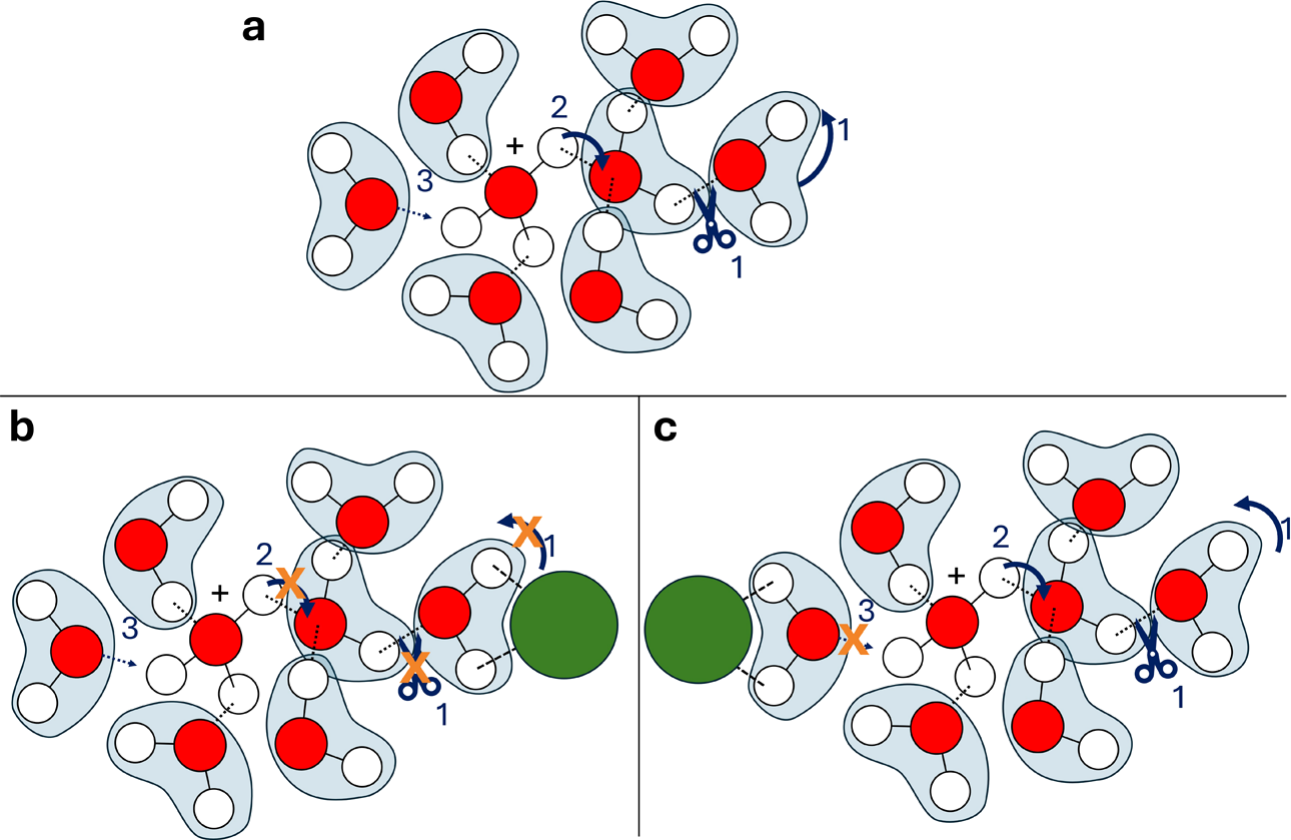
Schematic illustration to explain the Grotthuss mechanism (a) in infinite dilution as described by Agmon,^22^ and (b, c) suppressed by an ion. Water molecules in the hydration shell of ions are strongly bound, disturb the hydrogen-bond network, and therefore (b) block the proton hopping (step 2), or (c) omit the reorganization of the hydrogen-bond network (step 3), leading to backward hopping of the H^+^.

Since we assume a single rate-determining step involving free water and hydrated ion A, *r*_G_ can be described using second-order kinetics with a rate constant *k*_G_, eq 6

6 $$r_{G}=k_{G}\left\langle c_{A}c_{W}^{f}\right\rangle$$

where subscript *W* denotes water. The brackets in eq 6 indicate the possibility for preferential orientation within the solution, as is common for ions in electrolytes.^12^ Additionally, if the concentration of ions neither affects the average distance which the charge is transferred by the Grotthuss mechanism $\left(|\overset{®}{\Delta x}|\right)$ , nor its direction,^24^ $\overset{®}{\Delta x}$ and $\overset{®}{\Delta x^{0}}$ cancel out in eq 5. Then, by inserting eq 6 into eq 5, we find eq 7

7 $$\frac{\lambda_{A,G}}{\lambda_{A,G}^{0}}=\frac{\frac{k_{G}}{c_{H^{+}}}\left\langle c_{A}c_{W}^{f}\right\rangle }{\frac{k_{G}^{0}}{c_{H^{+}}^{0}}\left\langle c_{A}^{0}c_{W}^{f,0}\right\rangle }$$

Following our definitions, we define: *c*_*W*_^*f*^ = *c*_*W*_^*t*^ – *c*_*W*_^*b*^,^21^ where “*t*” denotes the total concentration of water. Additionally, we express the total concentration *c*_*W*_^*t*^ with reference to the concentration at infinite dilution *c*_*W*_^t,0^, introducing the term δ*c*_*W*_ that expresses the water concentration as a function of the ion concentration: δ*c*_*W*_ = *c*_*W*_^*t*,0^ – *c*_*W*_^*t*^. Inserting these definitions into eq 7 and noting that the water concentration at infinite dilution is equal to the total concentration (*c*_*W*_^*f*,0^ = *c*_*W*_^*t*,0^), we get eq 8

8 $$\frac{\lambda_{A,G}}{\lambda_{A,G}^{0}}=\frac{\frac{k_{G}}{c_{A}}\left\langle c_{A}\left(c_{W}^{t,0}-\delta c_{W}-c_{W}^{b}\right)\right\rangle }{\frac{k_{G}^{0}}{c_{A}^{0}}\left\langle c_{A}^{0}c_{W}^{t,0}\right\rangle }$$

Following the works and notation of Debye,^52^ as well as Kirkwood and Buff,^53^ we rewrite ⟨*c*_A_ c*_W_* ⟩ in terms of the average concentrations and radial distribution functions *g*_AW_, eq 9

9 $$\left\langle c_{A}c_{W}\right\rangle =g_{\mathrm{AW}}\left\langle c_{A}\right\rangle \left\langle c_{W}\right\rangle$$

In thermodynamic equilibrium, the radial distribution functions *g* are well-defined^54,55^ but during conductivity measurements, they are intrinsically disturbed as an applied field introduces preferential movement and orientation of ions. However, the electric fields applied during conductivity measurements ranging within few V/m^56^ are very small compared to the microscopic fields within electrolytes, resulting in negligible deviations between radial distribution functions in thermodynamic equilibrium and during conduction.^50,57^ Nevertheless, using thermodynamic radial distribution functions introduces some uncertainty. To highlight these uncertainties, we mark the distribution functions during conduction with a tilde *g̃*. Inserting eqs 9 into [Disp-formula eq8], we get eq 10

10b λA,GλA,G0=kGcA(g̃AWt⟨cA⟩⟨(cWt,0−δcW)⟩−g̃AWb⟨cA⟩⟨cWb⟩)kG0cA0g̃AW0⟨cA0cWt,0⟩(10a)=kG(g̃AWt⟨(cWt,0−δcW)⟩−g̃AWb⟨cWb⟩)kG0g̃AW0⟨cWt,0⟩(10b)

For the distribution functions, we note *g*_AW_^*t*^ ≈ 1 and *g*_AW_^0^ = 1, as water molecules in the first hydration shell of ions are explicitly not considered. Additionally, the rate constant is assumed to remain independent of the ion concentration (*k*_G_^0^ = *k*_G_). Hence, we find eq 11

11b λA,GλA,G0=⟨(cWt,0−δcW)⟩−g̃AWb⟨cWb⟩⟨cWt,0⟩(11a)=1−δcW+g̃AWbcWbcWt,0(11b)

The term δ*c*_*W*_ quantifies the reduced concentration of water as ion concentration increases and is exactly evaluable if concentration-dependent data of the electrolyte density is available. The term *g̃*_AW_^*b*^ *c*_*W*_^*b*^ quantifies the number of contacts between hydrated A and bound water occupying a reaction site (thus omitting the proton hopping there). To evaluate *g̃*_AW_^*b*^ *c*_*W*_^*b*^, we separate *c*_*W*_^*b*^ into parts for water being bound to the respective ions, *c*_*Wk*_^*b*^, by introducing the number of bound water molecules *h*_*k*_ per ion *k* ^21^

12 $$c_{W}^{b}=c_{\mathrm{WA}}^{b}+c_{\mathrm{WX}}^{b}$$

with

13 $$c_{\mathrm{Wk}}^{b}=h_{k}c_{k}$$

To account for the electrostatic interaction between *k* and *A*, we introduce the radial distribution functions of A and the counterions (both around A) and evaluate it for the contact (denoted by superscript “c”) of hydrated ions with a hydrated A. We get eq 14

14 $${\mathrm{g̃}}_{\mathrm{AW}}^{b}c_{W}^{b}={\mathrm{g̃}}_{\mathrm{AA}}^{c}h_{A}c_{A}+{\mathrm{g̃}}_{\mathrm{XA}}^{c}h_{X}c_{X}$$

As explained for eq 9, ${\mathrm{g̃}}_{\mathrm{AA}}^{c}$ and ${\mathrm{g̃}}_{\mathrm{XA}}^{c}$ account for the fact that equally charged ions, including their hydration shell, repel each other (and oppositely charged ions attract each other). Finally, inserting eq 14 into eq 11, we get eq 15

15 $$\frac{\lambda_{A,G}}{\lambda_{A,G}^{0}}=1-\frac{\delta c_{W}+{\mathrm{g̃}}_{\mathrm{AA}}^{c}h_{A}c_{A}+{\mathrm{g̃}}_{\mathrm{XA}}^{c}h_{X}c_{X}}{c_{W}^{t,0}}$$

Assuming an ideally mixed electrolyte implies *g̃*_AA_ = *g̃*_XA_ = 1 and we get the simplified model, eq 16

16 $$\lambda_{A,G}=\lambda_{A,G}^{0}\left(1-\frac{\delta c_{W}+\sum_{k}h_{k}c_{k}}{c_{W}^{0}}\right)$$

Theoretically, eqs 15 and 16 can become negative, which is unphysical and displays a relevant limitation of the model. Reasons for this limitation and methods to overcome it will be discussed in Section 3. For now, any negative values that may occur for λ_A,G_ are set to zero, eq 17

17 $$\lambda_{A,G}\geq 0$$

We extend OM with GM and compare it to the measurement data of strong acids and bases. The combined model is denoted as “OM + GM” to indicate that an OM always serves as the basis. We use the equations and parametrization suggested by Roger et al.^32^ and provide them in the Supporting Information for completeness.

The molar conductivity of the solution is the sum of the ionic molar conductivities of all ions inside the solution,^38^ eq 18

18 $$\Lambda =\lambda_{A}+\lambda_{X}$$

OM returns concentration-dependent ionic molar conductivities (λ_A_^OM^ and λ_X_^OM^) accounting for the electrophoretic and relaxation effects. GM returns a concentration-dependent contribution to the ionic molar conductivities (λ_A_^GM^) only for the part ascribed to the Grotthuss mechanism. The results of GM, so the reduced Grotthuss conductivity, do not feed back into OM. Thus, in OM, there is no distinction between the Vehicle and the Grotthuss mechanism. Hence, for X and A we get eq 19 ,b, respectively

19a $$\lambda_{X}=\lambda_{X}^{\mathrm{OM}}$$

19b $$\lambda_{A}=\lambda_{A}^{\mathrm{OM}}-\left(\lambda_{A,G}^{0}-\lambda_{A,G}\right)$$

The term inside the brackets in eq 20 describes the reduction of the ionic molar Grotthuss conductivity with ion concentration (according to GM) and is evaluated using eqs 15 or 16, respectively.

The values of the distribution functions at contact ${\mathrm{g̃}}_{\mathrm{XA}}^{c}$ and ${\mathrm{g̃}}_{\mathrm{AA}}^{c}$ are estimated using eqs 21 and 22

20 $${\mathrm{g̃}}_{\mathrm{kA}}^{c}=\exp\left(-\frac{w_{\mathrm{XA}}^{c}}{k_{B}T}\right)$$

21 $$w_{\mathrm{kA}}^{c}=\frac{z_{X}z_{A}e^{2}}{4\pi \varepsilon_{0}\varepsilon_{R}}\frac{1}{\sigma_{\mathrm{kA}}^{c}\left(1+\Gamma \sigma_{\mathrm{kA}}^{c}\right)}$$

where the screening parameter Γ is taken from the MSA, *z* is the valence, *e* is the elementary charge, *k*_B_ is the Boltzmann constant, *T* is the temperature, ε_0_ and ε_*R*_ are the vacuum and relative permittivity, respectively, and *k* ∈ {*X*, *A*}. σ_*kA*_^*c*^ is the distance between the centers of ion *k* and *A* at contact of the hydrated ions with radii σ_*k*_^*c*^ and σ_*A*_^*c*^, respectively. For σ_*k*_^*c*^, we take the distance from ion *k* to the first oxygen peak in the distribution functions evaluated with data from the literature (see Table 1). The resulting concentration-dependent values of ${\mathrm{g̃}}_{\mathrm{kA}}^{c}$ are shown in Figure 3. For the simplified GM (eq 16), ${\mathrm{g̃}}_{\mathrm{kA}}^{c}$ is unity for both ions in all acids and bases by definition. A parameter variation of σ_*k*_^*c*^ is shown in the Supporting Information.

**Figure 3:**
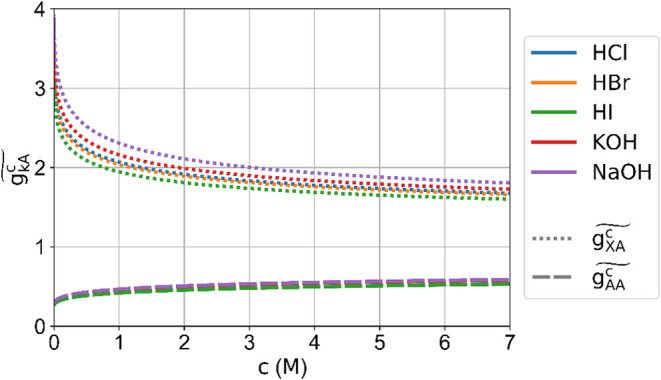
Distribution functions around a central ion A evaluated at the contact of hydrated ions. The value of ${\mathrm{g̃}}_{\mathrm{AA}}^{c}$ is always smaller than 1 due to repelling ion charges but increases with concentration. The value of ${\mathrm{g̃}}_{\mathrm{XA}}^{c}$ is always larger than 1 due to opposite ion charges but decreases with concentration.

**Table 1:** Parameterization Used within This Work^a^

ion	H^+^	Na^+^	K^+^	OH^–^	Cl^–^	Br^–^	I^–^
σ_*k*_^*c*^ [Å]	2.6 ^63^	2.43 ^64^	2.81 ^64^	2.8 ^65^	3.3 ^66^	3.4 ^62^	3.7 ^66^
λ_*k,G*_^0^/λ_*k*_^0^	0.90	0.00	0.00	0.85	0.00	0.00	0.00
*h*	3.0	2.2	1.6	3.0	2.4	2.3	2.0
*h* ^stat^	3.0 ^4^	2.4 ^62^	2.0 ^62^	3.0 ^4^	3.9 ^62^	3.8 ^62^	3.2 ^62^

a Radii of hydrated ions σ_*k*_^*c*^, fraction of the ionic molar conductivity assigned to the Grotthuss mechanism λ_*k,G*_^0^/λ_*k*_^0^, number of bound water molecules *h*, and static hydration numbers *h* ^stat^ reported by Jing et al.^62^ for comparison to our parameter *h*.

The Term δ*c*_*W*_ in eqs 15 and 16 is evaluated using eq 23 with mass density data from the literature^58–61^

22 $$\delta c_{W}=c_{W}^{t,0}-\frac{\rho -{(M}_{X}c_{X}+M_{A}c_{A})}{M_{W}}$$

where ρ is the electrolyte density and *M* denotes the molar mass. The parametrization used in this work is shown in Table 1. All equations for the Onsager-type conductivity model and the evaluation of the radial distribution functions at contact are shown in the Supporting Information.

## 3 Results and Discussion

In the following, we compare the Onsager-type conductivity model of Roger et al.^32^ based on the MSA (“OM”) and our model for the Grotthuss mechanism (“GM”, in addition to the OM) to experimental conductivity data from the literature. Figure 4 shows the molar conductivity as a function of the molar concentration *c* of strong binary electrolytes of exemplary acids (HCl, HBr, HI), bases (KOH, NaOH), and neutral salts (KCl, NaCl, NaBr). The neutral salts show a decrease in conductivity of up to 71 S cm^2^/mol going from infinite dilution to 4 M. Neutral salts are not affected by the contribution of GM and serve as a reference for the accuracy of OM. The bases and acids show a decline in molar conductivity of about 148 and 232 S cm^2^/mol, going from infinite dilution to 4 M, respectively. For the bases and acids, OM shows a similar trend as for the neutral salts while GM additionally decreases the modeled molar conductivity, improving the predictions. The simplified GM (eq 16) shows slightly higher molar conductivities than the complete model (eq 15) for acids and bases. The differences between the complete and the simplified GM are discussed in the Supporting Information in detail.

**Figure 4:**
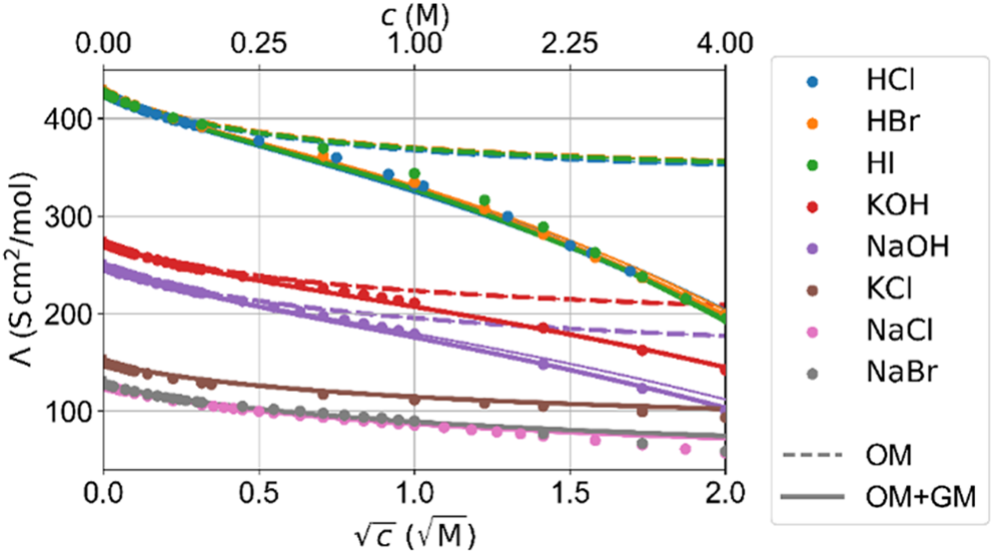
Molar conductivity of aqueous electrolytes from strong acids, bases, and neutral salts as a function of the concentration c compared to results from OM (Onsager-type conductivity model) and OM+GM (OM including the contribution developed here). The thin lines show the results using the simplified GM. Measurement data (dots) from the literature.^67–77^

Figure 5a shows a parameter variation of the number of bound water molecules *h*, which are varied by 50% with respect to the reference values (Table 1). Higher *h* values increase the effect of GM, leading to a more rapid decline in molar conductivity while lower *h* values decrease the effect. Although the hydration number is varied severely, a quantitative improvement of the prediction for the molar conductivities is achieved compared to OM in all cases. Figure 5b shows the variation of the molar conductivity that is assigned to the Grotthuss mechanism λ_G,A_^0^ in infinite dilution as depicted in Figure 1. λ_G,ref_^0^ denotes the (reference) values we assigned to the Grotthuss mechanism thus far. The minimum value of λ_G_^0^ results from the reduction of the total molar conductivity (λ_A_^0^) by the upper limit of the Vehicle mechanism (λ_A,V_^max^). The upper limit of λ_G_^0^ results from assigning 100% of the ionic molar conductivity of the H^+^ or OH^–^ to the Grotthuss mechanism. Increasing the fraction of the ionic molar conductivity that is assigned to λ_G_^0^ decreases the predicted molar conductivities using the Grotthuss model. The variation of λ_G,A_^0^ within the limits discussed during the derivation shows a small sensitivity compared to the variation of *h*.

**Figure 5:**
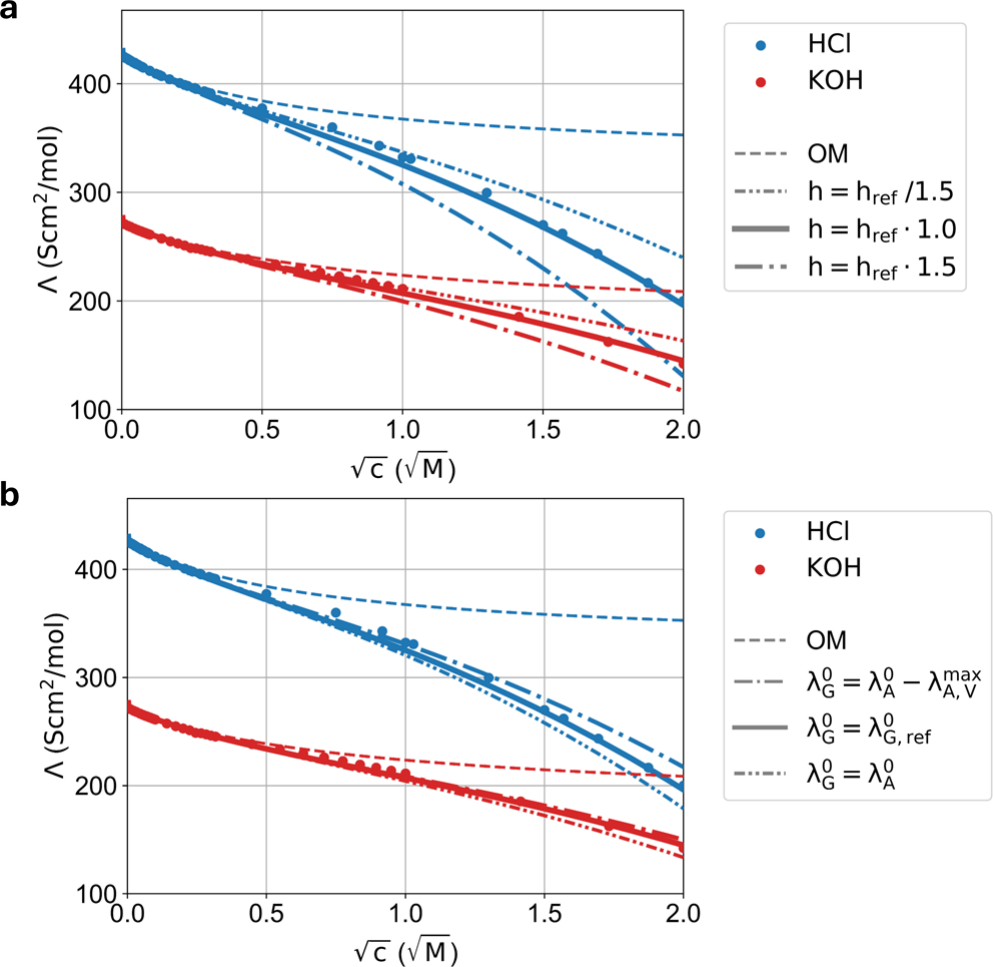
Parameter variation of (a) the hydration numbers *h*, and (b) the molar conductivity assigned to the Grotthuss mechanism. *h*_ref_ denotes the reference parametrization as shown in Table 1. λ_*G*,ref_^0^ denotes the reference parametrization as shown in Figure 1. Measurement data (dots) from the literature.^70,72–74^

Figure 6 shows the (absolute) conductivity of the discussed acids and bases as a function of the concentration on a linear scale up to 7 M. At concentrations of 7 M, the model limitation of eq 17 is almost reached for the acids. In this interval, the (measured) conductivities of all acids and bases show maxima. OM significantly overestimates the conductivity for concentrations larger than 2 M for acids (more than 145mS/cm; more than 25% too high) and bases (more than 60 mS/cm; more than 16% too high) and, unlike the measured conductivity, steadily increases. In contrast, OM+GM shows maxima for the conductivity of KOH and NaOH that quantitatively show good agreement with the measurement data. For the acids, a good agreement of model and experimental data is given up to 4 M. For higher concentrations, the modeled conductivity decreases significantly too fast.

**Figure 6:**
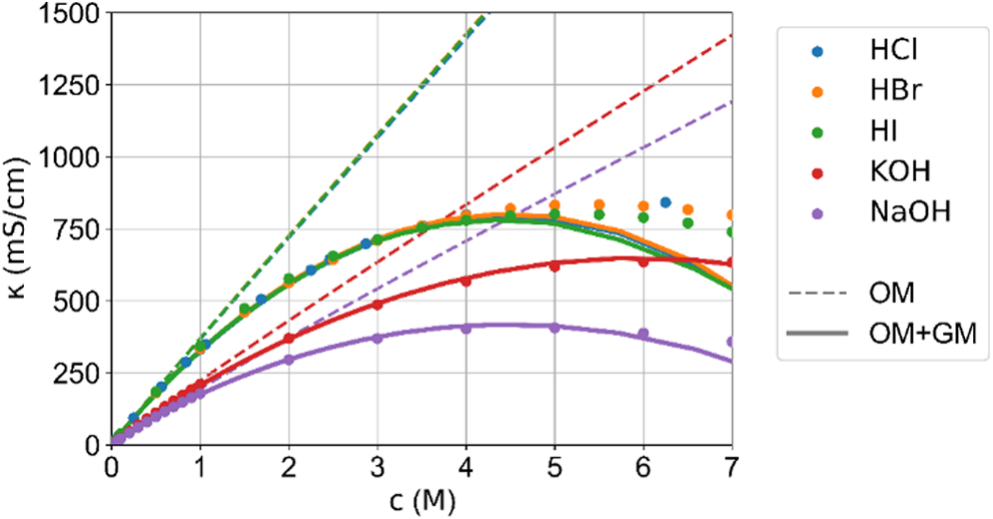
Conductivity of strong acids and bases as a function of the concentration c modeled with OM (the Onsager-type conductivity model) is compared to OM + GM (the model developed here) and to measurement data from the literature. For NaOH and KOH, the Grotthuss model depicts the maxima in the conductivity quantitatively correct. For the acids, the accuracy drastically decreases over 4 M. Measurement data (dots) from the literature.^70,72–77^

Figures 4–6 show that extending the traditional OM with GM allows us to accurately describe the molar conductivities of strong acids and bases. GM gives an explanation why the molar conductivity of (strong) acids and bases decreases faster than explained by traditional OM. Importantly, GM as well as the simplified GM (see Supporting Information) predict maximum conductivities of strong acids and bases for the first time without the requirement to consider association. The parameter variation illustrates that the Grotthuss model quantitatively improves the prediction of molar conductivities of acids and bases regardless of the exact values of both parameters. In particular, we showed that the model results are not sensitive to the exact values assigned to λ_G_^0^ as long as they are within theoretically discussed bounds (λ_A_^0^ – λ_A,V_^max^ ≤ λ_G_^0^ ≤ λ_A_^0^).^37,45^ The hydration numbers *h* depict more sensitive and physically complicated parameters. Their values and physical interpretation will be discussed in the following section.

The number of bound water molecules *h* introduced in this work quantifies the decreased probability of water molecules that are part of the hydration shell of ions to donate a hydrogen bond (or clear one). Hence, *h* may be interpreted as the number of water molecules bound to an ion *h* ^0^ weight with the probability to occupy a reaction side and the probability to omit a successful Grotthuss mechanism. *h* ^0^ may be interpreted by means of hydration numbers or coordination numbers. The hydration number, in contrast to the coordination number, incorporates an indication about the hydration strength.^62,78^ The hydration strengths in the first hydration shell of an ion may vary significantly between water molecules^66^ but generally decrease with ion size for alkali metal ions.^79,80^ The effect of hydration on the hydrogen-bond network is considered a local effect,^81^ not surpassing the first hydration shell.^82,83^ Additionally, hydration only shows a weak dependence on the counterions and concentration.^84^ This reasoning supports our assumption that the presence of ions locally decreases the probability of an effective Grotthuss mechanism. Consequently, our parameter *h* should be compared to hydration numbers rather than coordination numbers. Table 1 shows the static hydration numbers (*h* ^stat^) reported by Jing et al.^62^ and our parameter *h* for the ions considered in this work. Our parameter *h* is in general smaller than the respective reported static hydration number but shows a qualitatively similar trend to theirs, which is in line with our assumptions. An application of the hydration numbers reported by Jing et al.^62^ to our model is shown in the Supporting Information (Figure S2), still showing a reasonable accuracy. However, it should be noted that the exact values of hydration numbers and coordination numbers depend on their respective definitions, which are not consistent in the literature.^78^

The overestimation of the declining effect of the Grotthuss model at higher concentrations (especially for acids, see Figure 6) may be explained by an overestimation of bound water at higher concentrations, structural changes inside the electrolyte, such as ion association becoming dominant, or hopping mechanisms that are neglected in the model (additional reaction pathways). While hopping mechanisms with higher activation energies may be negligible in dilute systems, they can become dominant at higher concentrations.^28^ Considering additional reaction mechanisms with their respective activation energies could increase the applicability of the model but would introduce further parameters (including their uncertainties). Likewise, considering a concentration dependence of *h* may benefit the applicability at higher concentrations. After all, the limitations and uncertainties discussed, as well as those of the used OM, should be kept in mind: OM incorporates the ionic molar conductivity at infinite dilution and does not distinguish between the vehicle and hopping mechanism.

## 4 Conclusion

In this work, we developed a model (GM) that quantifies how the molar conductivity due to the Grotthuss mechanism decreases with the ion concentration. In GM, the Grotthuss mechanism is described by second-order reaction kinetics involving water molecules that can donate (or clear hydrogen bonds), which is in line with the literature. Thus, GM describes an effect not considered in traditional Onsager-type conductivity models (OM), which explain the decreasing molar conductivity of electrolytes only due to electrophoretic and relaxation effects. We evaluated GM as an extension of OM by comparing both to experimental data from the literature. GM explains the rapidly declining molar conductivities of strong acids and bases with increasing ion concentrations that could not be explained by traditional OM. With an optimized parametrization for the number of bound water molecules per ion, *h*, GM (as an extension for OM) enables excellent agreement with measurement data of strong acids and bases. The parameter *h* has a strong correlation with the hydration numbers of the respective ions. However, independent of the exact parametrization, the characteristic maxima in conductivity of acids and bases are qualitatively predicted for the first time in the context of OM without requiring association.

In future work, the GM may be validated for mixtures of salts with acids and bases. Additionally, GM may serve as a building block for models describing solid electrolytes like membranes, where the conductivity largely depends on the hydration state of the membrane. Lastly, addressing the distinction between vehicle and hopping mechanisms for Onsager-type models may reduce model uncertainties.

## Supplementary Material





## Data Availability

The data underlying this study, including scripts and code used to generate and analyze the results, are available in the published article and the Supporting Information.

## Notes

Implementation of all equations in Python files, including the creation of graphs shown in this work (ZIP)
Additional model results, including segmented model contributions and parameter variations, as well as equations constituting the Onsager-type conductivity model (PDF)
