# Supplementary material for: Complementing Onsager’s Conductivity Theory by Grotthuss Mechanism Mitigation via Ion-Induced Depletion of Hydrogen-Bond-Donating Water
Source: J Chem Theory Comput. 2026 Jul 4;22(14):7096–104. doi: 10.1021/acs.jctc.6c00901 (PMC13422001; doi:10.1021/acs.jctc.6c00901)
Supplement: Supplementary file 2 [file ct6c00901_si_002.pdf]

Supporting Information

to

Complementing Onsager's conductivity theory by Grotthuss mechanism mitigation via ion-induced depletion of hydrogen-bond-donating water

Benjamin Janotta* [a], Maximilian Schalenbach [a], Hermann Tempel [a], Wei-Hsuan Hung [b], Rüdiger-A. Eichel[a,c,d]

[a] Fundamental Electrochemistry (IET-1), Institute of Energy Technologies, Forschungszentrum Jülich, Wilhelm-Johnen-Straße, 52425 Jülich, Germany

[b] Institute of Materials Science and Engineering, National Central University, No. 300, Zhong-da Rd., Zhongli District., Taoyuan City 320, Taiwan, ROC

[c] Institute of Physical Chemistry, RWTH Aachen University, 52062 Aachen, Germany

[d] Faculty of Mechanical Engineering, RWTH Aachen University, 52056 Aachen, Germany

** Corresponding author:* b.janotta@fz-juelich.de

Keywords: Grotthuss mechanism, conductivity, electrolytes, strong acids and bases, Onsager conductivity

Additional model results

Figure S1 shows the effects of the decreased water concentration δc_W, the decreased concentration of free water due the counterion X, $\delta c_{W,X}$, as well as species A, $\delta c_{W,A}$, on the Grotthuss conductivity λ_G. δc_W decreases the Grotthuss conductivity slightly for HCl and KOH. The effect of water bound to the respective ions ($\delta c_{W,A}$ and $\delta c_{W,X}$) shows a larger effect on λ_G than δc_W. The effect of $\delta c_{W,X}$ is smaller for the simplified model than for the complete

model, while $\delta c_{W,A}$ shows the opposite trend. In total, these differences between the simplified and the complete model almost cancel out.

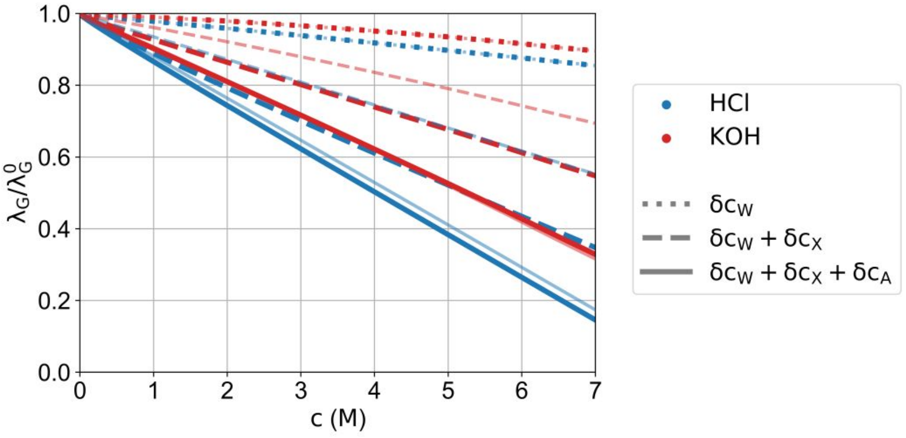

Figure S1: Normalized Grotthuss conductivity as a function of the concentration c. δc_W quantifies the effect of the changed water concentration with acid or base concentration. $\delta c_{W,X}$ quantifies the effect of water bound to ions X, and $\delta c_{W,A}$ denotes the effect of water bound to ions A. Thin lines indicate the simplified model.

Figure S2 shows the molar electrolyte conductivity for using the static hydration numbers given by Jing et al. for our parameter h. While the bases still show a good overlap with measurement data from the literature, the modelled conductivity of the acids is too low.

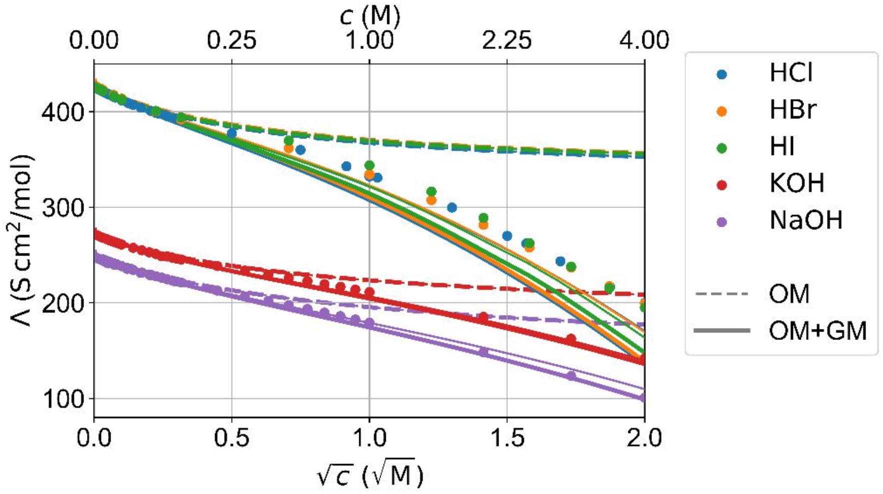

Figure S2: Molar conductivity of aqueous electrolytes from strong acids, bases, and neutral salts compared to predictions from the OM and OM+GM using the hydration numbers reported by Jing et al.[1] for our parameter h. The thin lines show the results using the simplified GM. Measurement data from the literature[2–8].

Figure S3 shows the distribution functions around a central ion A evaluated at contact of hydrated ions as a function of the concentration for a variation of the radii of hydrated ions σ_k^c. For higher radii (increased by 0.1 Å, so an increased ion distance of 0.2 Å), the values of the distribution functions evaluated at contact is closer to 1 than before.

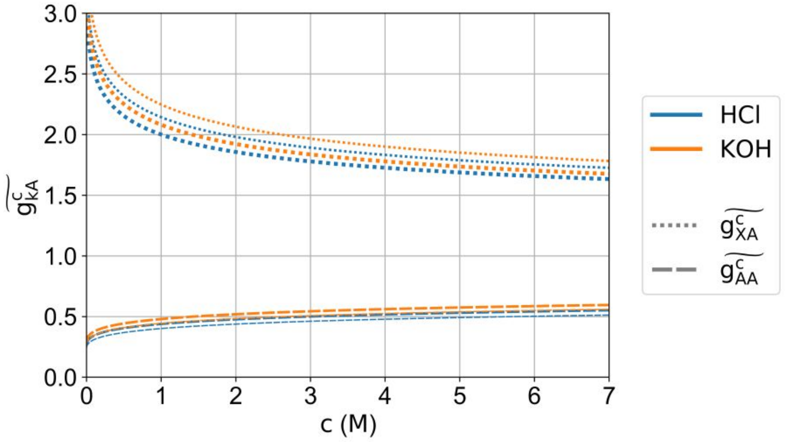

Figure S3: Distribution functions around a central ion A evaluated at contact of hydrated ions. The radii of hydrated ions σ_k^c (given in Table 1 in the main article) are varied by ± 0.1 Å, so the distance of the ionic centers at contact of the hydration shell is varied by ± 0.2 Å. Thin lines correspond to decreased radii, thick lines to increased radii.

Onsager conductivity

In this work, the molar conductivity of electrolytes is modelled based on an Onsager-type conductivity model developed within the Mean Spherical Approximation by Roger et al.[9]. For the derivation of and details on the MSA, the reader is referred to the literature[9,10]. Here, we show the equations used for the model denoted as "OM" in the main article. The equations are pure repetition from the literature with limited explanation. The following text coincides to large amounts with our previously published Supporting Information to the article "Buffering effects of supporting electrolytes on pH profiles in electrochemical cells"[11].

At infinite dilution, the velocity of an ion is proportional to an (external) electric field acting on it, where the proportionality is given by the mobility. At finite concentrations, the ion is additionally affected by other ions in its ionic atmosphere and the drag due to motion of the solvent (electrophoretic force). The ionic atmosphere of an ion (which is on average oppositely charged to it) moves in the opposite direction of the ion due to its charge. Thus, the center of charge of the ion and its ionic atmosphere are separated, leading to a relaxation force which opposes the (external) electric field partially and reduces its velocity.

Electrophoresis (superscript "eph") and relaxation (superscript "rel") reduce the effective conductivity of the ions in solution. The ionic equivalent conductivity of an ion as a function of both forces can be expressed by Eq. 1:

$$\lambda_k = \lambda_k^0 \left(1 + \delta_k^{eph}\right)\left(1 + \delta_k^{rel}\right) \tag{1}$$

Here, the final equations to calculate the conductivity in multi-ion electrolytes based on single ion properties at infinite dilution are given for the sake of completeness.

The electrophoretic effect δ_k^{eph} is given by Eq. 2:

$$\delta_k^{eph} = -\frac{k_\mathrm{B}T}{3\pi\, z_k D_k^0 \eta^0}\left(\Xi_k + \frac{\pi N_\mathrm{A}}{4}\sum\nolimits_j c_j z_j \sigma_j^2 - \frac{\pi N_A}{6}\sum\nolimits_j c_j \sigma_j^3 \Xi_k\right), \qquad (2)$$

where k_B is the Boltzmann constant, N_A Avogadro's constant, T is the temperature, z the valence, c the concentration, D_k^0 the diffusion coefficient at infinite dilution, η^0 the solvent viscosity, and $\Xi_k$is defined by Eq. 3:

$$\Xi_k = \frac{\Gamma z_k}{1+\Gamma\sigma_k} + \frac{\zeta\sigma_k}{1+\Gamma\sigma_k}. \qquad (3)$$

The screening length Γ is defined by the implicit function Eq. 4:

$$\Gamma^2 = \frac{e^2}{4\, k_B T \varepsilon}\sum_j^s c_j\, N_A \left(\frac{z_j - \zeta\sigma_j^2}{1+\Gamma\sigma_j}\right)^2, \qquad (4)$$

where:

$$\zeta = \frac{\pi}{2\Omega\Delta}\sum_j \frac{c_j\, N_A \sigma_j z_j}{1+\Gamma\sigma_j}, \qquad (5)$$

and

$$\Omega = 1 + \frac{\pi}{2\Delta}\sum_j^s \frac{c_j\, N_A \sigma_j^3}{1+\Gamma\sigma_j}. \qquad (6)$$

Eq. 4 to Eq. 6 must be solved numerically due to their implicit nature.

The relaxation forces of one ion depend on all other ions in solution. δ_k^{rel} is given by Eq. 7:

$$\delta_k^{rel} = -\frac{\kappa^2 e_k}{3}\sum_{p=1}^s \mathrm{X}_k^p \sum_{j=1}^s \sum_{i=1}^s \frac{t_j \mathrm{X}_j^p I_i\left(e_i u_i' - e_j u_j'\right)}{e_i e_j\left(u_i' + u_j'\right)} \frac{\sinh\left(\kappa\sqrt{q_p}\,\sigma_{ij}\right)}{\kappa\sqrt{q_p}\,\sigma_{ij}}\,\Theta_\mathrm{ij}. \qquad (7)$$

κ is the inverse Debye screening length with:

$$\kappa = \sqrt{\sum_j \frac{N_A c_j e_j^2}{\varepsilon k_B T}}. \qquad (8)$$

σ_{ij} are the distances of closest approach between ions i and j, Eq. 9:

$$\sigma_{ij} = (\sigma_i + \sigma_j)/2. \qquad (9)$$

I_k denotes the relative ionic strength of k, Eq. 10:

$$I_k = \frac{c_k\, e_k^2}{\sum_j c_j e_j^2} \qquad (10)$$

$\overline{u'}$ is the mean mobility defined by Eq. 11:

$$\overline{u'} = \sum_{j}^{s} I_j\, u'_j, \tag{11}$$

where the prime denotes that the mobility is divided by Avogadro's constant: $u'_k = u_k/N_A$.

Using the notation above, the transport numbers t_k at infinite dilution can be expressed by Eq. 12:

$$t_k = \frac{I_k u'_k}{\overline{u'}} \tag{12}$$

χ_k^p and N_p are defined by Eq. 13:

$$\chi_k^p = \sum_{j}^{s} \frac{N_p u'_k}{{u'_k}^2 - \alpha_p^2} \tag{13}$$

and Eq. 14:

$$\frac{1}{N_p^2} = \sum_{j}^{s} \frac{t_j {u'_j}^2}{\left({u'_k}^2 - \alpha_p^2\right)^2} \tag{14}$$

where the α_p are the roots of Eq. 15:

$$-2\overline{u'}\alpha \sum_{j}^{s} \frac{t_j}{\left({u'_j}^2 - \alpha^2\right)^2} = 0. \tag{15}$$

To find the roots efficiently, Onsager showed that they follow Eq. 16:

$$0 = \alpha_1^2 < {u'_1}^2 < \alpha_2^2 < \dots < \alpha_s^2 < {u'_s}^2 \tag{16}$$

Θ is given by Eq. 17:

$$\Theta_{ij} = \int_{\sigma_{ij}}^{\infty} r\ \exp\left(\kappa\sqrt{q_p}\,\sigma_{ij}\right) h_{ij}^0 dr \tag{17a}$$

$$\approx -\frac{e_i e_j \kappa\sqrt{q_p}\,\sigma_{ij} \exp\left(\kappa\sqrt{q_p}\,\sigma_{ij}\right)}{4\pi\varepsilon k_B T\left(\kappa^2 q_p + 2\Gamma\kappa\sqrt{q_p} + 2\Gamma^2 - 2\Gamma^2 Y\right)}, \tag{17b}$$

where the substitute Y is defined by Eq. 18:

$$Y = \frac{\sum_j^s \frac{c_j N_A z_j^2}{\left(1+\sigma_j\Gamma\right)^2} \exp\left(\kappa\sqrt{q_p}\,\sigma_j\right)}{\sum_j^s \frac{c_j N_A z_j^2}{\left(1+\sigma_j\Gamma\right)^2}} \tag{18}$$

The only (fitting) parameters necessary in the MSA are the ionic radii, given in Table 1 in the main article.

References

1. Jing, Z. *et al.* Hydration of Alkali Metal and Halide Ions from Static and Dynamic Viewpoints. *The journal of physical chemistry letters* **14,** 6270–6277; 10.1021/acs.jpclett.3c01302 (2023).

2. Darken, L. S. & Meier, H. F. Conductances of Aqueous Solutions of the Hydroxides of Lithium, Sodium and Potassium at 25°. *Journal of the American Chemical Society* **64,** 621–623; 10.1021/ja01255a046 (1942).

3. Marsh, K. N. & Stokes, R. H. The conductance of dilute aqueous sodium hydroxide solutions from 15° to 75°. *Aust J Chem* **17,** 740–749; 10.1071/CH9640740 (1964).

4. DeWane, H. J. & Hamer, W. J. Electrochemical data. Part 10 - Electrolytic conductivity of aqueous solutions of the alkali metal hydroxides. Electrolytic conductivity of aqueous solutions of the alkali metal hydroxides, 1968.

5. Haase, R., Sauermann, P.-F. & Dücker, K.-H. Leitfähigkeiten konzentrierter Elektrolytlösungen. *Zeitschrift für physikalische Chemie* **47,** 224–245; 10.1524/zpch.1965.47.3_4.224 (1965).

6. Owen, B. B. & Sweeton, F. H. The Conductance of Hydrochloric Acid in Aqueous Solutions from 5 to 65°. *Journal of the American Chemical Society* **63,** 2811–2817; 10.1021/ja01855a091 (1941).

7. Shedlovsky, T. THE ELECTROLYTIC CONDUCTIVITY OF SOME UNI-UNIVALENT ELECTROLYTES IN WATER AT 25°. *J. Am. Chem. Soc.* **54,** 1411–1428; 10.1021/ja01343a020 (1932).

8. Hamer, W. J. & DeWane, H. J. Electrolytic conductance and the conductances of the halogen acids in water. Institute for Basic Standards, 1970.

9. Roger, G. M., Durand-Vidal, S., Bernard, O. & Turq, P. Electrical conductivity of mixed electrolytes: Modeling within the mean spherical approximation. *The journal of physical chemistry. B* **113,** 8670–8674; 10.1021/jp901916r (2009).

10. Blum, L. Mean spherical model for asymmetric electrolytes. *Molecular Physics* **30,** 1529–1535; 10.1080/00268977500103051 (1975).

11. Janotta, B., Schalenbach, M., Turiaux, M., Tempel, H. & Eichel, R. A. Buffering effects of supporting electrolytes on pH profiles in electrochemical cells. *Scientific reports* **15,** 32458; 10.1038/s41598-025-18219-z (2025).

12. Simonin, J.-P. & Verweij, W. A Simplified Mean Spherical Approximation Model for the Description of Activity Coefficients in Electrolyte Mixtures. *Ind. Eng. Chem. Res.* **61,** 13265–13274; 10.1021/acs.iecr.2c02039 (2022).
